# Decomposition-Based Multiobjective Evolutionary Algorithm for Community Detection in Dynamic Social Networks

**DOI:** 10.1155/2014/402345

**Published:** 2014-03-02

**Authors:** Jingjing Ma, Jie Liu, Wenping Ma, Maoguo Gong, Licheng Jiao

**Affiliations:** Key Laboratory of Intelligent Perception and Image Understanding of Ministry of Education of China, Xidian University, Xi'an 710071, China

## Abstract

Community structure is one of the most important properties in social networks. In dynamic networks, there are two conflicting criteria that need to be considered. One is the snapshot quality, which evaluates the quality of the community partitions at the current time step. The other is the temporal cost, which evaluates the difference between communities at different time steps. In this paper, we propose a decomposition-based multiobjective community detection algorithm to simultaneously optimize these two objectives to reveal community structure and its evolution in dynamic networks. It employs the framework of multiobjective evolutionary algorithm based on decomposition to simultaneously optimize the modularity and normalized mutual information, which quantitatively measure the quality of the community partitions and temporal cost, respectively. A local search strategy dealing with the problem-specific knowledge is incorporated to improve the effectiveness of the new algorithm. Experiments on computer-generated and real-world networks demonstrate that the proposed algorithm can not only find community structure and capture community evolution more accurately, but also be steadier than the two compared algorithms.

## 1. Introduction

Many real-world complex systems can be represented as complex networks. Networks could be modeled as graphs, where nodes (or vertices) represent the objects and edges (or links) represent the interactions among these objects. The area of complex networks has attracted many researchers from different fields such as physics, mathematics, biology, and sociology. Besides a number of distinctive properties such as the small-world effect, the right-skewed degree distributions, and network transitivity that many networks seem to share, community structure is another important property in complex networks [1]. Qualitatively, a community is defined as a subset of the graph nodes which densely connect with each other and sparsely connect with the rest of the networks [2, 3].

In recent years, dynamic networks have become an increasing interest due to their great potential in capturing natural and social phenomena over time [4], such as the analysis of the evolution of research communities within and across academic disciplines and the analysis of mobile subscriber networks [5]. In the previous study, a two-step approach is widely adopted. Firstly, a static analysis is applied to the snapshots of the network at different time steps, and then community evolution is introduced afterward to interpret the change of communities over time [6]. However, data from real-world networks are ambiguous and subject to noise. Under such scenarios, if an algorithm extracts community structure for each time step independently, it often results in community structures with a high temporal variation [7].

Some recent studies have attempted to unify the processes of community extraction and evolution by using certain heuristics, such as regularizing temporal smoothness. This idea comes from a new kind of clustering concept called evolutionary clustering which has been proposed to capture the evolutionary process of clusters in temporal data [8]. This framework assumes that the structure of clusters significantly changing in a very short time is less desirable, and so it tries to smooth out each cluster over time.

Evolutionary clustering could be regarded as evolutionary multiobjective optimization (EMO). The optimization problems with only one objective are called single-objective optimization problems, and those with more than one objective are called multiobjective optimization problems (MOPs). The main purpose of EMO is to deal with multiobjective optimization problems by evolutionary computation. It has become a hot topic in the area of evolutionary computation. By simultaneously optimizing two or more than two objectives, multiobjective optimization evolutionary algorithm (MOEA) can acquire a set of solutions considering the influence of all the objective functions. Each of those solutions cannot be said to be better than the others and corresponds to a tradeoff between those different objectives.

Community detection in dynamic networks is a problem which can naturally be formulated with two contradictory objectives and consequently be solved by an MOEA. Nevertheless, how to make the best use of MOEA to detect community structures in dynamic networks has not been fully investigated. Motivated by these, a decomposition-based MOEA for community detection in dynamic social networks (DYN-DMLS) is proposed. DYN-DMLS employs the framework of MOEA/D [9] to simultaneously optimize the modularity [2] and normalized mutual information [10], which quantitatively measures the quality of the community partitions and temporal cost, respectively. The problem-specific knowledge is incorporated in genetic operators and local search to improve the effectiveness and efficiency of our method. The uniqueness of DYN-DMLS lies in the following three aspects.

(a) It is the first time to apply the framework of MOEA/D to detect community structure of dynamic networks. MOEA/D is applied as the framework of the proposed algorithm. It optimizes N scalar subproblems simultaneously instead of a single one. It has been proved to be effective in solving MOPs by a lot of literature [9, 11–13] (http://cswww.essex.ac.uk/staff/qzhang/webofmoead.htm). Each subproblem is a single-objective optimization problem. To describe the advantage, we take DYN-MOGA [14] as the comparison. DYN-MOGA is significant because it may be the first trial that adopts MOEA to detect dynamic networks. Properly speaking, its optimization adopts the genetic algorithm with NSGA-II [15] dealing with multiple solutions. During the selection of solutions, to escape getting trapped into the local optimization, NSGA-II introduces the crowding distance to enhance the sparse part of the solution set, which relies on the assumption that the sparse helps to jump out of the local optimization. This is partly applicative and does not always give an expected result. MOEA/D decomposes the original problem into several subones. The scope is wide at the very beginning. When detecting dynamic networks, each calculation would involve different networks. That means that the preference to either objective may be dynamic. MOEA/D is more flexible and capable at this point. It provides an extensive possibility to approach the ideal solution before selection, rather than reforming the solution set as NSGA-II. The experimental results in Section 5 show that DYN-DMLS outperforms DYN-MOGA obviously.

(b) Problem-specific genetic operators and a local search operator are designed for community detection in dynamic networks. Problem-specific genetic operators make use of neighborhood information to enhance the performance of crossover and mutation. The neighborhood information comes from the topology of network. Gene mutates among the neighboring alleles resulting in the fact that each offspring is a meaningful code. Then, uniform crossover would be surely safe in exchanging genes between two meaningful parents. The genetic operators avoid unnecessary search burden significantly. Moreover, label propagation [16] working as the local search operator could find a better solution effectively and efficiently. Label propagation also utilizes the topological information. Not only the connectivity but also the strength in numbers is under consideration. The quantity of neighbors with the same community ID plays a key role in adjusting the current individual's clustering. The principle meets the definition of community, in which members are closed to each other. Label propagation could produce a plenty of solutions like this in a very short time for each subproblem. Then, single-objective optimization could quickly find the best. In general, label propagation as local search strategy is effective for making use of the topology of network, while it is efficient for its perfect cooperation with MOEA/D.

(c) NMI and modularity work as objective functions perfectly, which is proven by our experiments. As mentioned in [8], evolutionary clustering decomposes the dynamic optimization problem into two objectives, snapshot quality and history cost. Snapshot quality is well studied on static networks, which is measured by famous modularity here. The most important is how to measure history cost. Based on the assumption of time smoothing (dramatic shifts between networks of two consecutive time steps are undesirable), a computable distance between two kinds of clustering is needed. Normalized mutual information (NMI), a well-known entropy measure in information theory, just works here. A number of researches on static networks have regarded NMI as the evaluation metric to measure the accuracy of results compared with ground truth. Naturally, the calculated objects of NMI could be replaced by the results of two consecutive time steps. Therefore, the dynamic of networks could be described by NMI step by step. Some discussions on the effect of NMI as time smoothing are given in the experiment part. It is clear that time smoothing presented by NMI performs significantly. In addition, the time symmetry of DYN-DMLS is also discussed. We made an analysis from the view of MOEA and provided a simple experiment to show the time symmetry which is not so good.

Experiments on computer-generated and real-world networks show the performance of our algorithm. Compared to the state-of-the-art algorithms, our algorithm has the ability to discover the community structure and its evolution more accurately.

The remainder of this paper is organized as follows: Section 2 reviews several state-of-the-art MOEAs and introduces related work of community detection in dynamic networks.  Section 3 describes the proposed algorithm in detail sequentially.  Section 4 presents the experimental study. Finally, concluding remarks are given in Section 5.

## 2. Related Work

MOEA is the base of our work. It is so basic that a brief introduction is enough. There are many famous MOEAs that have been proposed in recent years. For instance, NSGA-II [15], SPEA2 [17], MOEA/D [9], MOPSO [18], and so forth are the state-of-the-art approaches. NSGA-II uses a nondominated sorting and crowding distance to generate the nondominated solution set. SPEA2 is an improved elitist multiobjective evolutionary algorithm that employs an enhanced fitness assignment strategy compared to its predecessor SPEA. In SPEA2, new techniques for archive truncation and density-based selection are proposed. MOPOS is an extension of PSO to handle multiobjective problems. In our precious work [19–23], MOEAs have been successfully applied to handle community detection problems. MOEA/D will be introduced in Section 4.

Dynamic network is the topic mainly discussed in this paper. Dynamic networks could be analyzed in many kinds of aspects, for example, the tracing of communities, the prediction, and the evolution. In [24], a multiple objective evolutionary algorithm has been proposed by us on dynamic networks. In [25], there is a summary. Next we will introduce some significant related events in this field.

Existing methods for analyzing communities and their temporal evolution in dynamic networks can be divided into two classes. For the first class, communities and their evolutions are studied separately (usually community structures are independently extracted at each time step and then in retrospect). For the second class, communities and their evolutions are studied in a unified framework where the temporal smoothness is incorporated into analyzing communities, in order to make community structure more appropriate.

The first method to detect dynamic network structures is proposed by Hopcroft et al. [26]. They identify natural communities in each snapshot by hierarchical clustering based on similarity and match them among different snapshots. Their approach can trace the lifetime of communities, but hierarchical clustering hardly works well. In [4], Palla et al. give a thorough study on real dynamic social networks. Statistically, they conclude some principles correlated to the community size and the community life time; for example, larger communities may last longer. Their conclusions are realistic but highly rely on the static community detection algorithm CPM. The above two are typical two-stage approaches, which calculate the static networks first and then infer the correlations among them. The deviation of structure and time leads to unexpected fluctuation. Therefore, the result has its limits.

To overcome the fluctuation of two-stage approaches, the framework of evolutionary clustering was introduced by Chakrabarti et al. [8]. It is a unified framework to obtain clusters from snapshot quality and history cost simultaneously. The expected result has a high snapshot quality (it should fit the current network) and a low history cost (it should be similar to the previous one). It becomes the basic principle soon which many researchers apply and modify. Our DYN-DMLS is also in this category.

An evolutionary spectral clustering approach, proposed by Chi et al. [27], first employs the framework of temporal smoothness to cluster real blog data. They use graph cut as the metric for measuring community structure and community evolution. This method integrates temporal smoothness in the overall spectral clustering to obtain more stable and consistent clustering results, which are less sensitive to short-term noises and adaptive to long-term cluster drifts. Tang et al. [28] use a joint matrix factorization method to discover the community evolution. These methods try to maximize cluster accuracy, with respect to incoming data of the current time step, and minimize clustering drift from one time step to the successive one. In order to optimize both of these two competing objectives, it is necessary to control the degree of the user's preference towards either the snapshot cost or the temporal cost. Thus, Folino and Pizzuti proposed a multiobjective genetic algorithm to discover communities in dynamic networks by employing genetic algorithm [14]. Facetnet [29] proposed by Lin et al. relies on formulating the problem in terms of nonnegative matrix factorization. As the monotonic decrease of cost function, it promises to converge to an optimal solution with a low time complexity. But Facetnet needs a fixed community quantity, while Kim and Han's method [30] could handle the situation with variable community quantities. The concept of nanocommunity is applied to describe the dynamic of networks. The evolving, forming, and dissolving happen at the level of particles. Moreover, with cost embedded technique smoothing the network data, density-based clustering method dividing the network, and some deposition mapping local clusters, the evolution of the network would be revealed. But particle and density-based method is sensitive to the parameter used in the clustering step.

DYN-MOGA [14] proposed by Folino and Pizzuti can detect arbitrary quantities of network communities and is independent of parameters. It is an extension of multiobjective evolutionary algorithm. The two objectives naturally correspond to the snapshot quality and history cost. The optimization is a searching process for the individuals with a relatively high snapshot quality and a relatively low history cost. More recently, Lancichinetti and Fortunato propose consensus clustering [31]. It has a good performance on both the static and dynamic networks. Consensus clustering could extract stable results from a series of partitions. Therefore, it could be combined with any existing method in a self-consistent way, enhancing considerably both the stability and the accuracy of the resulting partitions [31]. For improving all kinds of classic static clustering algorithms, it is like standing on the shoulders of giants and being able to see further. It is also suitable to handle dynamic networks. The stable result generated by consensus clustering from several consecutive time steps could evaluate the network structure of a certain time window. They showed the great performance of consensus clustering on monitoring the evolution of community structure in real temporal networks.

Our DYN-DMLS has a close relationship with DYN-MOGA. It is certain that DYN-MOGA is involved in the comparison. While consensus clustering is the latest algorithm representing the current level of this field, we also take it into the comparison.

There is a large amount of researches focusing on other properties of dynamic network besides community structure. Ahmed and Karypis tried to mine the evolution of conserved relational states from dynamic networks in their new paper [32]. Their work aims at finding the evolution path of detected relational states called evolving induced relational state (EIRS). It is similar to a set of communities with temporal relations. The entities involved may change slightly at every time step, but the main body remains stable. The changing states may lead to a better understanding of network dynamics. Kunegis et al. have another point of view on dynamic networks. In [33], they make efforts to validate that, in the spectral evolution model, the growth of large networks can be described by a change of the spectrum while the corresponding eigenvectors remain constant. Then, based on this experimental fact, they proposed two link prediction algorithms aiming at predicting where new edges will appear in a growing network. In [34], the three-way data and its evolution were paid much attention. In [35], mining temporal network models were focused on and the models are the base of the dynamic analysis. In [36], a model was presented, which can describe the collective blogging behavior on popular incidental topics.

## 3. Proposed Algorithm

A dynamic network (DN) can be modeled as a sequence of graph *G*_*t*_(*V*_*t*_, *E*_*t*_); that is, DN = {*G*_1_, *G*_2_,…, *G*_*t*_,…}, where *G*_*t*_ is the graph representing a snapshot network at time step *t*, *V*_*t*_ is a set of objects in *G*_*t*_, called nodes or vertices, and *E*_*t*_ is a set of links each of which connects two objects of *V*_*t*_. A community in a network is a group of vertices having a high density of edges within them and a lower density of edges between groups. Let *CR*_*t*_ denote the set of community partitions for *G*_*t*_, let *C*_*t*_^*i*^ denote a community composing the community partition *CR*_*t*_: that is, *CR*_*t*_ = {*C*_*t*_^1^, *C*_*t*_^2^,…*C*_*t*_^*i*^,…, *C*_*t*_^*k*^}, and let *k* denote the number of communities in *CR*_*t*_.

Assuming *G*_*t*_ has *n* nodes, the adjacency matrix *W*_*t*_ (*W*_*t*_ ∈ *R*_+_^*n*×*n*^) is used to represent the link between nodes in *G*_*t*_, where *w*_*i*,*j*_ represents the element at the *i*th row and *j*th column of *W*_*t*_. If there is an edge from node *i* to node *j*, *w*_*i*,*j*_ = 1; otherwise *w*_*i*,*j*_ = 0.

### 3.1. Framework of MOEA/D-Based Dynamic Community Detection

As mentioned above, community detection in dynamic networks is a problem which can naturally be formulated with two contradictory objectives. One objective is the community quality at the current time. The other objective is the temporal cost, which measures the distance between two community structures at consecutive time steps. In this paper, the framework of MOEA/D [9] is applied to optimize the two conflicting objectives in dynamic networks.

MOEA/D maintains a population *X* = {*x*_1_,…, *x*_*N*_} throughout the optimization process. At each generation, the population is evolved in the following steps. First, a unique solution *x* ∈ *X* is assigned to each subproblem, which is called its representative. Then, *N* subpopulations are constructed, each for a subproblem. Second, for the *i*th subproblem, two parents are selected such that one is *i*th subproblem and the other is from the whole population. In such a way, a very wide range of child solutions could be generated due to the dissimilarity among these parent solutions. Therefore, the exploration ability of the search could be enhanced. Then crossover, mutation, and local search are applied to the parents to generate an offspring *y*_*i*_ to update the current solutions to its neighboring subproblems. By repeating this procedure for all subproblems, a new population *Y* = {*y*_*i*_,…, *y*_*N*_} is generated. Finally, an external population (EP), which is used to store nondominated solutions found during the search, is maintained. The framework of the MOEA/D-based community detection algorithm for dynamic networks is given in Algorithm 1.

### 3.2. Initialization

To start MOEA/D, the decomposition of the original problem is needed. MOEA/D initially decomposes the MOP into *N* single-objective subproblems. The subpopulation of a subproblem associated with a weight vector *λ* ^*i*^ is composed of the representatives of the *T* subproblems whose associated weight vectors are the *T* closest (in terms of Euclidean distance) weight vectors to *λ* ^*i*^, where *T* is the size of subpopulation. As stated in [9], the optimal solution of the *i*th subproblem should be close to that of the *j*th subproblem if *λ* ^*i*^ is close to *λ* ^*i*^. Thus, one new solution, generated for each subproblem, is employed to update that of the other subproblem in its subpopulation. Here, we employ the Tchebycheff approach [37] as a decompositional technique. The *j*th single-objective optimization subproblem is defined as follows:

(1) minimize gj(x ∣ λj,z∗)=max⁡1≤i≤2{λij ∣ fi(x)−zi∗}subject  tox∈Ω,

where z* = (*z*_1_*,*z*_2_*)^*T*^ is the reference point, that is, *z*_i_* = max⁡{*f*_*i*_(*x*) | *x* ∈ *Ω*}, for each *i* = 1,2, *λ* ^*j*^ = (*λ*_1_^*j*^,*λ*_2_^*j*^)^*T*^ is the weight coefficients of subproblem, and ∑_1≤*i*≤2_ *λ*_*i*_^*j*^ = 1 and *λ*_*i*_ = (*i* − 1)/(*N* − 1). To obtain the evenly distributed Pareto-optimal solutions, it is important to choose proper weights. In our approach, we adopt uniformly distributed weight vectors. In the aggregation approaches, such as MOEA/D, the *λ* ^*i*^ is mainly used for decomposing an MOP into single-objective subproblems by adding different weights to the objectives. This is the initialization to the objective problem.

In order to solve each subproblem, the network at the first time step should provide a kind of community structure as the initialization to time smoothing. Because there is no history information at the first time step, the network can be clustered without time smoothing. Therefore, in Step 1 of Algorithm 1, it is a single optimization on the community quality objective. The memetic community detection algorithm (Meme-Net) by ourselves [38] is adopted. Meme-Net optimizes modularity to obtain a satisfying structure. All the subproblems share this structure as their base for the second time step. In the coming time steps, each subproblem would develop by itself. Note that MOEA/D has turned the original problems into several sub-ones. Here, each individual in the population would correspond to one subproblem. Assume that the original problem is decomposed into *N* single-objective optimization subproblems. A population of initial solutions with size *N* would be generated. Meme-Net completes the initialization to the object of study.

### 3.3. Representation

**Figure 1:**
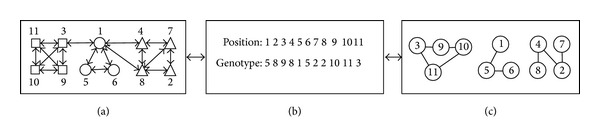
Illustration of the LAR. (a) A network modeled as a graph; (b) the LAR of one possible genotype; (c) the community structure of the genotype.

Following our previous work in [38], in this paper, we use locus-based adjacency representation (LAR) proposed in [39] and employed by [40] for multiobjective clustering. In this graph-based representation, an individual *g* in the population consists of *n* genes, in which each gene corresponds to a node in the network and *n* denotes the total number of nodes in this network. And each gene *i* can take an arbitrary allele value *j* in the range {1,2,…, *n*}, which means a link between node *i* and *j* existing in the corresponding graph *G*. This also means nodes *i* and *j* might be in the same community in the network. The main advantage of this representation is that the number *k* of clusters is automatically determined by the number of components contained in an individual and the decoding step. In addition, the decoding process can be done in a linear time, which illustrates that this encoding schema is very effective for community detection. The LAR and the corresponding encoded genotype are shown in Figure 1.

The locus-based adjacency encoding scheme has several major advantages for our task. Firstly, it is unnecessary to fix the number of communities in advance, as it is automatically determined in the decoding step, which is an important feature to address the real-world networks with no prior knowledge. Secondly, some standard crossover operators such as uniform, one-point, or two-point crossover can be employed in this representation, which effortlessly implements merging and splitting operations of communities on individuals and also maintains the remainder of the partitioning. Finally, the genetic representation contains all possibilities of connected subgraphs, which guarantees that a better community structure can be obtained by maximizing the modularity.

### 3.4. Objective Functions

As mentioned above, under the framework of temporal smoothness, we need two objective functions to quantitatively measure the quality of the communities and temporal cost, respectively. A quantitative definition, network modularity, proposed by Girvan and Newman [1], is one of the most popular quality functions to assess the goodness of the partitioning. We use modularity to measure how well the cluster structure represents the current network partition. Another quantitative definition, normalized mutual information (NMI), is a similarity measure proved to be reliable by Danon et al. [10]. We use NMI to estimate the similarity between the current community structure and the previous one.

The modularity criterion is based on the intuitive idea that a random graph does not exhibit cluster structure, while possibly there is cluster structures that is revealed by the comparison between the actual density of edges in a subgraph and the density which one would expect to have in the subgraph if the vertices of the graph were attached regardless of community structure [25]. This expected edge density depends on the chosen null model, that is, a copy of the original graph keeping some of its structural properties but without community structure [25]. Modularity can then be written as follows:

(2) $$\begin{array}{c} Q=\overset{k}{\underset{c=1}{\sum }}\left[\frac{l_{c}}{m}-{\left(\frac{d_{c}}{2m}\right)}^{2}\right], \end{array}$$

where *k* is the number of clusters, *l*_*c*_ is the number of links inside cluster *c*, *m* is the total number of links in the network, and *d*_*c*_ is the total degrees of the vertices in cluster *c*. The first term of summand in (2) is the fraction of edges inside cluster *c*, and the second term represents the expected fraction of edges that would be in the random graph with the same community divisions. If the number of within-community edges is no better than that of the random, we will get *Q* = 0, while the value *Q* = 1, which is the maximum, indicates a strong community structure [1]. The higher the modularity *Q* is, the better the partition obtained is.

The second objective is NMI, which is a well-known entropy measure to evaluate how similar the community structure *CR*_*t*_ is with the previous clustering *CR*_*t*−1_ [10]. Given two partitions *A* and *B* of a network, let *C* be the confusion matrix whose element *C*_*ij*_ is the number of nodes of community *i* of the partition *A* that are also in the community *j* of the partition *B*. The normalized mutual information NMI(*A*, *B*) is defined as

(3) $$\begin{array}{c} \text{NMI}\left(A,B\right)=\frac{-2\sum_{i=1}^{C_{A}}\sum_{j=1}^{C_{B}}C_{ij}\log\left(C_{ij}N/C_{i.}C_{.j}\right)}{\sum_{i=1}^{C_{A}}C_{i.}\log\left(C_{i.}/N\right)+\sum_{j=1}^{C_{B}}C_{.j}\log\left(C_{.j}/N\right)}, \end{array}$$

where *C*_*A*_ and *C*_*B*_ denote the number of clusters in the partitioning *A* and *B*, respectively. *C*_*i*._ is the sum of the elements of *C* in row *i*, *C*_.*j*_ is the sum of the elements of *C* in column *j*, and *N* is the number of nodes. If *A* is identical to *B*, NMI(*A*, *B*) = 1. If *A* and *B* are completely different, NMI(*A*, *B*) = 0. Otherwise, NMI(*A*, *B*) ∈ (0,1). In order to make the current communities and the previous communities as similar as possible, we need to maximize NMI.

### 3.5. Problem-Specific Operators

#### 3.5.1. Uniform Crossover

**Figure 2:**
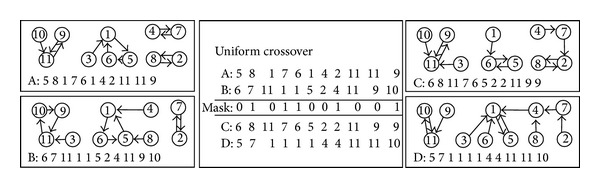
Illustration of uniform crossover. A uniform crossover on parents *A* and *B* is employed to generate the two children *C* and *D*.

In order to maintain the effective connections of the nodes in the child individual, uniform crossover is employed as the crossover operator in our method. Unlike one-point and two-point crossover, the uniform crossover enables the parent chromosomes to contribute to the gene level rather than the segment level and can generate any combination of alleles from the two parents [40]. Due to the biased initialization, if a gene *i* contains a value *j*, then the edge (*i*, *j*) exists, and each individual in the population is safe. Given two safe parents, individuals *A* and *B*, uniform crossover is performed on them to get the child individuals *C* and *D*. An example of crossover can be seen in Figure 2.

#### 3.5.2. Mutation

For the mutation, we adopt the neighbor-based mutation [41], in which the possible values of an allele are restricted to the neighbors of gene *i*. The neighbor-based mutation guarantees that, in a mutated child, each vertex is linked only with one of its neighbors. This can avoid the useless exploration of the search space. In the mutation operator, we randomly select some genes and assign other randomly selected adjacent nodes to them which effectively guarantees the generation of a safe mutated child.

#### 3.5.3. Local Search

According to many researches [16, 42, 43], the network structure itself can provide some crucial information about communities. The information is local and the process is efficient. In [16], Raghavan et al. proposed label propagation method to detect large network communities efficiently. It is constructed on the direct analysis of the network structure without any objective function. A unique label is initially assigned to each node. Then the iteration begins with each node turning to have the same label with its most neighbors. It would not stop until no label change is going to happen, which means the community structure is stable. The advantage includes the efficiency and the independence of objective functions or similarity metrics. The disadvantage concerned most is the fluctuation of the result. Though it may not always converge to the same result, the idea of utilizing the neighborhood is meaningful. Therefore, we considered taking it as our local search strategy.

Why could label propagation be the local search strategy here? Firstly, it is localized and quick by making use of the neighborhood information. Based on a given partition, the interaction between members' labels will provide a membership adjustment which may lead to some improvement. Label propagation imitates the process of communication in the real world. It is consistent with the definition of community. Secondly, though label propagation has a low stability, it still could contribute to the optimization process. As local search is a trail for better solutions, failure is acceptable. The effect of label propagation would be indicated within the iterations because it gets more chance to show. Thirdly, because MOEA/D works well in the global search, the input of the local search is nearly of high quality. It will enhance the stability of label propagation in some degree. The flow of local search is shown in Algorithm 2.

According to the feature of the MOEA/D, each subproblem is a single-objective optimization problem. Therefore, a better solution can be obtained by a local search procedure in optimizing corresponding single-objective problem.

In order to use the prior knowledge about relations between nodes, the local search strategy is based on the neighbor nodes. There is an obvious intuition that a node tends to be in the same community with most of its neighbors. In other words, if most of a node's neighbors are in the *i*th community, the node will be in the *i*th community with a high probability. Therefore, we should find the labels of all the neighbors of the node and record the nodes with the label whose number is the biggest among the neighbors. Then we randomly select one from these recorded nodes to replace the original node. It will not result in merging or splitting communities when moving this node from one community to the other one.

### 3.6. Solution Selection

MOEA/D decomposes the original problem into several sub-ones. Each subproblem is a single-objective optimization and provides one solution at the end of each time step. Subproblems exchange information within their neighborhood. Though it is a single-objetive optimization, dominant relationship is still implicated. The solution which is nondominated with both its own subpopulation and neighborhood would be reserved. These solutions form the nondominated solution set. The decomposition of the original problem supports the diversity of MOEA, while the dominant relationship with subpopulations and neighborhood pushes the solution set moving to Pareto front. The front is supposed to contain all the nondominated solutions theoretically. But in real world it is hard to realize. As the optimization of modularity and NMI are nondeterministic polynomial, we cannot identify whether the generated solutions by MOEA/D within limited generations are the optimal solution or not. MOEA/D makes efforts to approach Pareto front.

In this paper, modularity density [44] is used to select the best tradeoff solution from the dominant solution set. Modularity density is a quantitative measure for evaluating the partition of a network into communities based on the concept of average modularity degree, which has confirmed its effectiveness. Let an undirected graph *G* = (*V*, *E*) with |*V*| = *n* vertices and |*E*| = *e* edges. The adjacent matrix of the graph is *A*. Given a partition *Ω* = {*V*_1_,…, *V*_*M*_} of the graph, where *V*_*i*_ is the vertex set of subgraph *G*_*i*_ for *i* = 1,…, *M*. The modularity density (also called *D* values) is defined as

(4) $$\begin{array}{c} D=\overset{M}{\underset{i=1}{\sum }}\frac{L\left(V_{i},V_{i}\right)-L\left(V_{i},\bar{V_{i}}\right)}{\left|V_{i}\right|}, \end{array}$$

where *L*(*V*_*i*_, *V*_*i*_) = ∑_*m*∈*V*_*i*_,*n*∈*V*_*i*__ *A*_*mn*_ means the internal degrees of the subgraph *G*_*i*_; $L\left(V_{i},{\bar{V}}_{i}\right)=\sum_{m\in V_{i},n\in \bar{V_{i}}}A_{mn}$, which means the external degrees of the subgraph *G*_*i*_; each summand means the ratio between the difference of the internal and external degrees of the subgraph *G*_*i*_ and the size of the subgraph. The larger the value of *D* becomes, the more accurate the partition is. Thus the solution we select as the best is the one with maximum modularity density *D* from the nondominated solution set.

## 4. Experimental Study

In this section, we evaluate the effectiveness of the proposed decomposition-based multiobjective evolutionary algorithm with local search for community detection in dynamic networks (termed as DYN-DMLS for short) on two synthetic networks and three real-world networks. The compared algorithms include DYN-MOGA [14] which is the only existing dynamic multiobjective community detection algorithm, DYN-DMLS without the local search strategy (termed as DYN-DMEA), and consensus clustering [31] which is a different dynamic approach from MOEA. Consensus clustering could work with any static community detection algorithms. And many classic algorithms [45, 46] have been tested.

As to the performance metric, in the case that we have the ground truth for each time step, we directly adopt a similarity measure, normalized mutual information (NMI) [10, 47], to estimate the similarity between the true partitions and the detected ones. Note that the performance metric NMI is different from the objective function NMI. The former one calculates the difference between the current result and the ground truth, while the latter one calculates the difference between two consecutive time steps. The definition of NMI is introduced in detail in Section 4.4. In addition, the box plot [48] is used to illustrate the statistical distribution of the values of NMI for all compared algorithms based on 30 independent runs on most datasets. The box plot uses the median, the approximate quartiles, and the lowest and highest data points to convey the level, spread, and symmetry of the distribution of data values. In a notched box plot the notches represent a robust estimate of the uncertainty about the medians for box-to-box comparison. Symbol “+” denotes outliers.

The experiments are performed on an Intel Core2 Duo CPU machine with 1.98 GHz and 1.99 GB RAM. The parameter settings are as follows. The population size *N* is 100 and the number of generations is 300. The crossover operator and mutation operator are the same in the three algorithms, where crossover rate = 0.8 and mutation rate = 0.2. In MOEA/D, the neighborhood size is set to be 15. In the following experiments, the reported data are the statistical results based on 30 independent runs on each dataset.

Lancichinetti and Fortunato's consensus clustering needs a static algorithm as the base. In this study, we choose consensus clustering with label propagation method (LPM) [16] and order statistics local optimization method (OSLOM) [49] for comparison. The thought of LPM is applied in our local search strategy, while consensus clustering with OSLOM has been tested in [31], which works well in finding time evolution of clusters in real-world network. The two are more comparable than the rest.

Note that it is hard to determine which ground truth each consensus result corresponds to. This is due to the fact that several time steps are calculated together to produce a result representing the general state of the network structure during the time window. To make the comparison clear, we prepared a strategy for the consensus clustering determining the ground truth of its results. The strategy is to make the consensus algorithm running in a similar way to DYN-DMLS. In DYN-DMLS, the result of each time step is determined mainly by two aspects. One is the current network and the other is the previous one. Similarly, for consensus clustering algorithm we took *r* = 2 (which means every two consecutive time steps produce a result). And the ground truth of the result between *t* and *t* + 1 would be assigned to the (*t* + 1)th time step. Then, it is easy to determine the ground truth to calculate the evaluation metric with. Different strategies may lead to different results. To emphasize *r* = 2, the two methods would be marked as consensus-LPM-2 and consensus-OSLOM-2. In addition, the value of consensus run is set to 5, which means that the algorithm would collect 5 partitions for each time step and find the consensus among them. The threshold for pruning the consensus matrix is set to 0.5 as default.

### 4.1. Experiments on Synthetic Datasets

In order to evaluate the ability of our approach to successfully detect the community structures for dynamic networks, we use benchmark datasets. Benchmark networks take *z* as the parameter to control each node's connections with other communities. The higher the value of *z* becomes, the more confusing the network structures are. When *z* is higher than 8, usually it is considered as that there is not any community in the network. In our experiment, the benchmark parameter *z* varies from 5 to 8 and each *z* corresponds to a set of networks with 10 time steps.

Two kinds of benchmarks are involved. The first is the GN benchmark [1], which has a fixed quantity of community size. Each network has 128 nodes and 4 communities. Each community has 32 nodes. The dynamic is just the member exchanging between the original four communities. Between every two consecutive time steps each community has 10% nodes involved in the dynamic. The benchmark is marked as SYN-FIX.

The second is modified by Kim and Han [30] from the SYN-FIX, marked as SYN-VAR. It consists of a set of networks which contain the forming and dissolving of communities and the attaching and detaching of nodes. It also contains 10 timestamps and each one has 256 nodes. Its structure is changing in a more complex way. At the first time step the network has four communities. Then, at each time step, every one of the four original communities would update 16 nodes (16 nodes leave and another 16 join in). The number of communities is also changing. From start to end, the community quantity is changing as the sequence (4,5, 6,7, 8,8, 7,6, 5,4). The creation of a new community is the combination of four sets of 8 nodes. Each set separately comes from one of the four original communities. Each new community contains 32 nodes and lasts for 5 timestamps before its nodes return to their original position. We run our algorithm on this dynamic network to show the ability of capturing the splitting and merging of communities.

The involved comparison algorithms include DYN-DMEA which is the version of DYN-DMLS without local search, DYN-MOGA, consensus clustering with OSLOM (consensus-OSLOM-2), and consensus clustering with LPM (consensus-LPM-2). As consensus clustering [31] is an excellent method for detecting complex network structures and it is a totally different framework from the evolutionary algorithm, it is worth to make a comparison between the proposed algorithm and the consensus clustering algorithm.

#### 4.1.1. Results on SYN-FIX

In this study, we generate the datasets under four different levels by setting *z* = 5,6, 7,8, where the community structures gradually change from clear to fuzzy. The first time step is ignored here for a better view of the dynamic process. Each of the comparison algorithms needs a static run at the first time step. Latter figures are all displayed like this.

**Figure 3:**
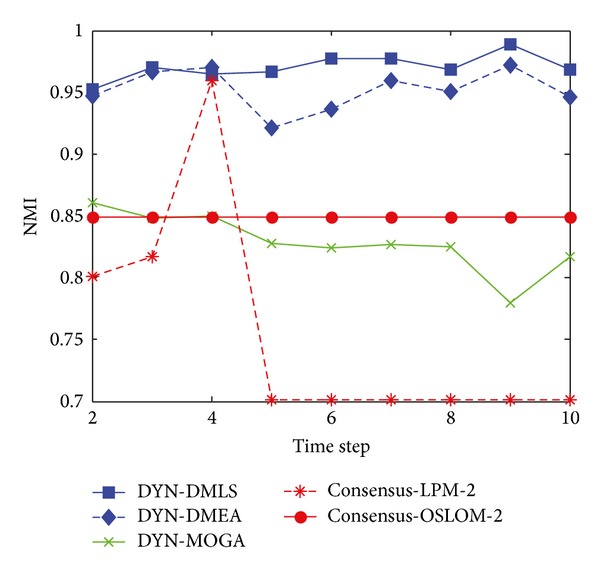
NMI results of the five algorithms on the SYN-FIX dataset with *z* = 5.

Figure 3 shows the statistical average NMI values of time steps from 2 to 10 with *z* = 5. Among the three MOEAs, our DYN-DMLS performs best. Its results keep stable at a high level. Without local search, DYN-DMEA's results get lower slightly. Both of them are better than DYN-MOGA. However, the two based on consensus clustering have a totally different tendency from each other. Consensus-LPM-2 gets a great fluctuation, while consensus-OSLOM-2 stays absolutely stable. The reason may be that consensus clustering relies much on the static algorithm chosen. Their results are a little low. But actually each single run of LPM or OSLOM on a single time step nearly gets the same clustering with ground truth. The combination of two consecutive time steps may cause a shift leading the final result to some middle state. Here the correspondence of consensus results with ground truth is kind of rough.

**Figure 4:**
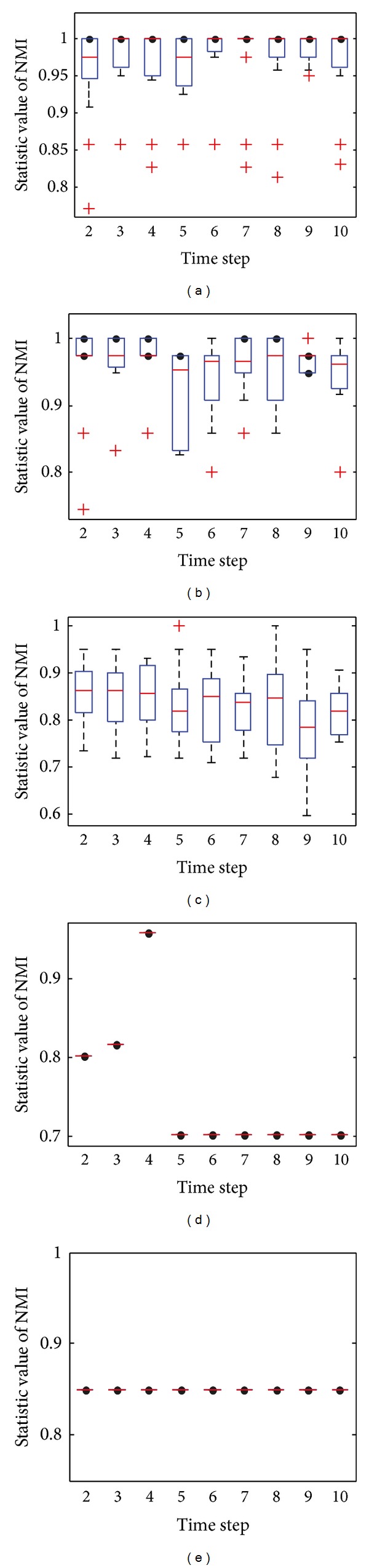
The box plots to illustrate the distribution of NMI at each time step when *z* = 5. (a) The box plot for DYN-DMLS; (b) the box plot for DYN-DMEA; (c) the box plot for DYN-MOGA; (d) the box plot for consensus with LPM; (e) the box plot for consensus with OSLOM.

Figure 4 shows the box plots of the value of NMI with respect to the ground truth at each time step over 30 independent runs with *z* = 5. Figures 4(a) and 4(b) clearly show that almost all of the values of NMI obtained by DYN-DMLS and DYN-DMEA are equal to 1, and DYN-DMLS is more stable than the DYN-DMEA. However, the values obtained by DYN-MOGA are not stable enough compared to the other two algorithms, as is shown in Figure 4(c). Therefore, DYN-DMLS is the most stable in the three algorithms. For consensus clustering, the result is not satisfying. The curve of consensus-OSLOM-2 is a straight line showing that the single run on each time step has the same result. Actually, OSLOM could detect static structures just the same as ground truth, but the consensus results turn to have a fixed deviation from ground truth. Consensus-LPM-2 performs worse.

**Figure 5:**
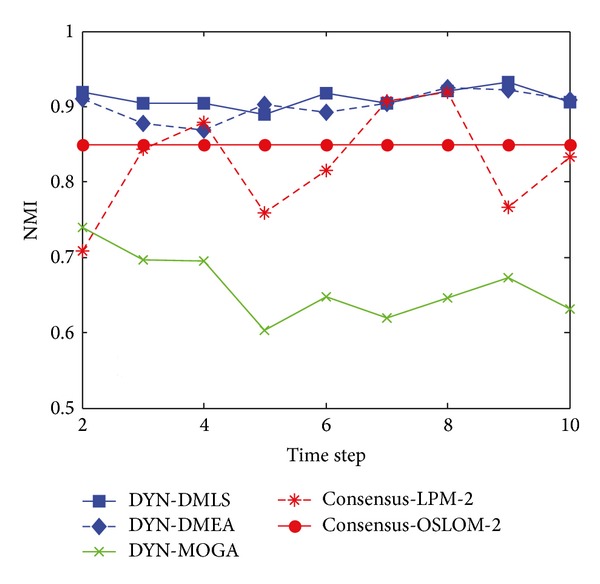
NMI results of the five algorithms on the SYN-FIX dataset with *z* = 6.

Figure 5 shows the statistical average value of NMI when *z* = 6. It is similar to that when *z* = 5. The average value of NMI obtained by DYN-DMLS and DYN-DMEA is close to 1, while DYN-DMLS outperforms DYN-DMEA. However, it is obvious that DYN-MOGA cannot find a more accurate community structure than the first two algorithms. Results of all three MOEAs are lower than those when *z* = 5. Consensus-LPM-2 is still of high variation and consensus-OSLOM-2 is also of high stability. Their results are lower than DYN-DMLS.

**Figure 6:**
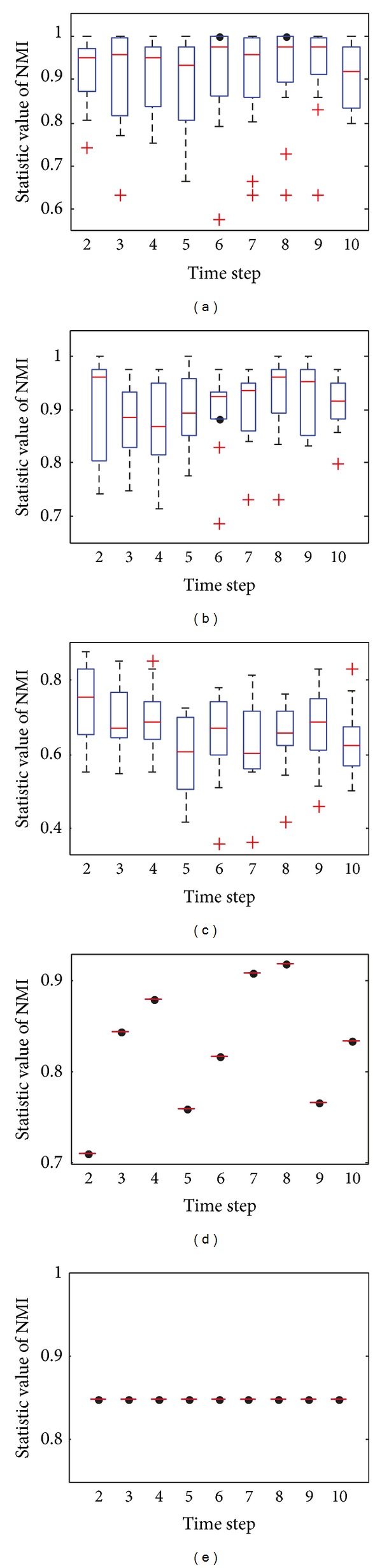
The box plots to illustrate the distribution of NMI at each time step when *z* = 6. (a) The box plot for DYN-DMLS; (b) the box plot for DYN-DMEA; (c) the box plot for DYN-MOGA; (d) the box plot for consensus with LPM; (e) the box plot for consensus with OSLOM.

Figure 6 shows the box plots to illustrate the distribution of the value of NMI, when *z* = 6. It can be seen clearly from Figure 6 that all the average values of NMI obtained by DYN-DMLS are above 0.9 over 30 independent runs while those obtained by DYN-DMEA range around 0.9 and those obtained by DYN-MOGA range from 0.6 to 0.75. Consensus clustering shows its stability again. The repeated run seems to have no meaning for it. Consensus clustering with OSLOM is better than that with LPM.

**Figure 7:**
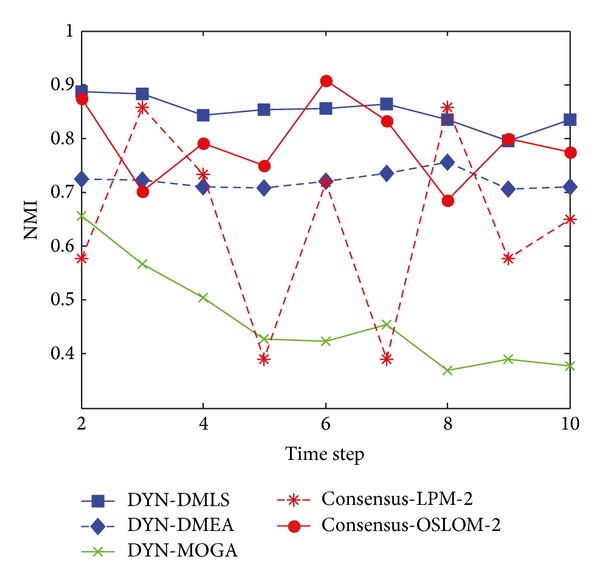
NMI results of the five algorithms on the SYN-FIX dataset with *z* = 7.

Figure 7 shows the statistical average value of NMI with *z* = 7. The network is so confused that the result turns lower and lower. It can be seen clearly that the average value of NMI obtained by DYN-DMLS is around 0.9. Without local search, DYN-DMLS just reaches 0.7. However, results obtained by DYN-MOGA are less than 0.7. Therefore, DYN-DMLS can find the most accurate community partition in the three algorithms. For consensus clustering, the degree of results lowering with the raising of *z* is little. Consensus-OSLOM-2 narrows the gap numerically, while consensus-LPM-2 still varies heavily due to the fact that LPM produces different structures in comparison with OSLOM.

**Figure 8:**
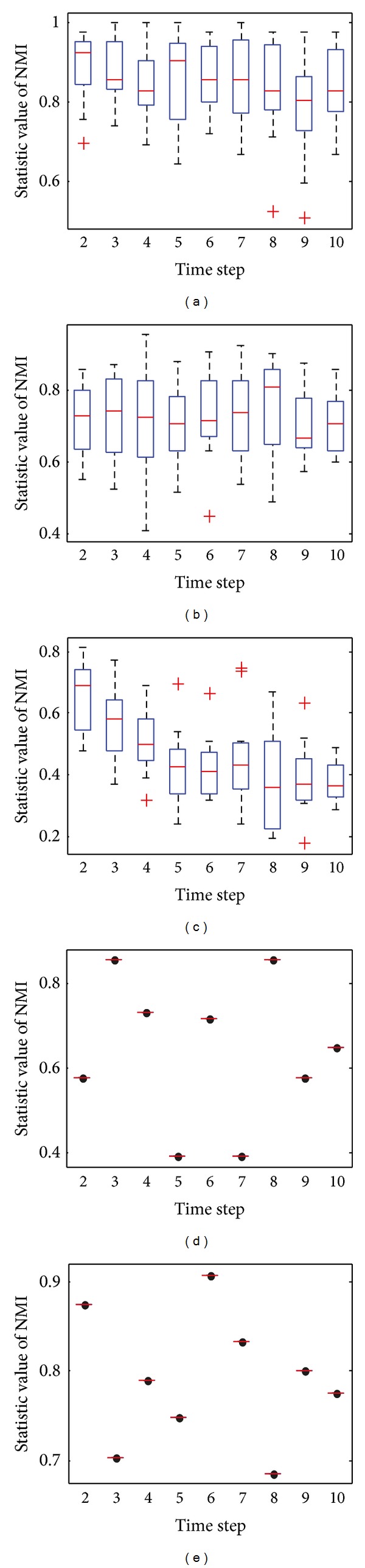
The box plots to illustrate the distribution of NMI at each time step when *z* = 7. (a) The box plot for DYN-DMLS; (b) the box plot for DYN-DMEA; (c) the box plot for DYN-MOGA; (d) the box plot for consensus with LPM; (e) the box plot for consensus with OSLOM.

Figure 8 shows the box plots to illustrate the distribution of the value of NMI, when *z* = 7, where the community structures become fuzzy and evolve relatively unstable over time. It can be seen clearly from Figure 8 that the majority of NMI values obtained by DYN-DMLS are above 0.8, and those obtained by DYN-DMEA range below 0.8 at the majority of time steps, while those obtained by DYN-MOGA range below 0.7 all the time. Therefore, it is obvious that the stability of DYN-DMLS is the best, DYN-DMEA is the second, and DYN-MOGA is the worst in the three algorithms. Consensus with OSLOM shows some fluctuation between time stamps but still converges as expected. Its result range is relatively high. Consensus with LPM varies heavily as usual and its range is wider and lower than that of consensus with OSLOM.

**Figure 9:**
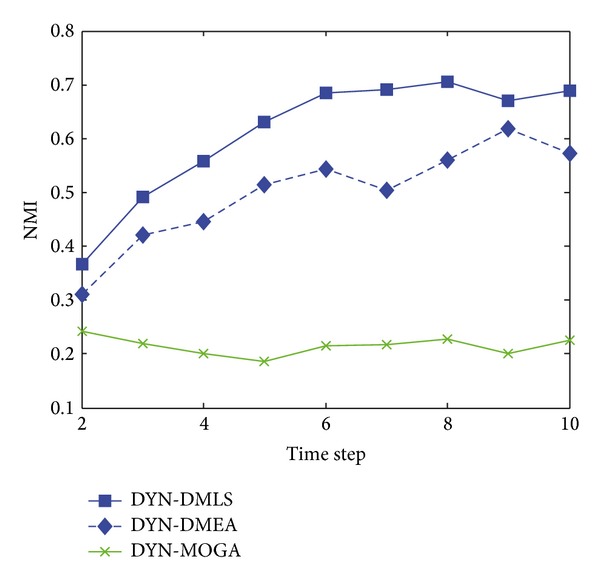
NMI results of the three algorithms on SYN-FIX dataset with *z* = 8.

Figure 9 shows the statistical average value of NMI, when *z* = 8. Now the networks are a complete mess. In this situation, though DYN-DMLS hardly detects the community structure effectively, it still improves the result over time. It is the same tendency with DYN-DMEA, while DYN-MOGA just stays at a low level without any vitality. Therefore, the community structure found by DYN-DMLS is the best in the three algorithms. Unfortunately, consensus clustering could not provide any result. Actually the reason is not clear. It may be relevant to the network. On SYN-VAR when *z* = 8, consensus clustering works well.

**Figure 10:**
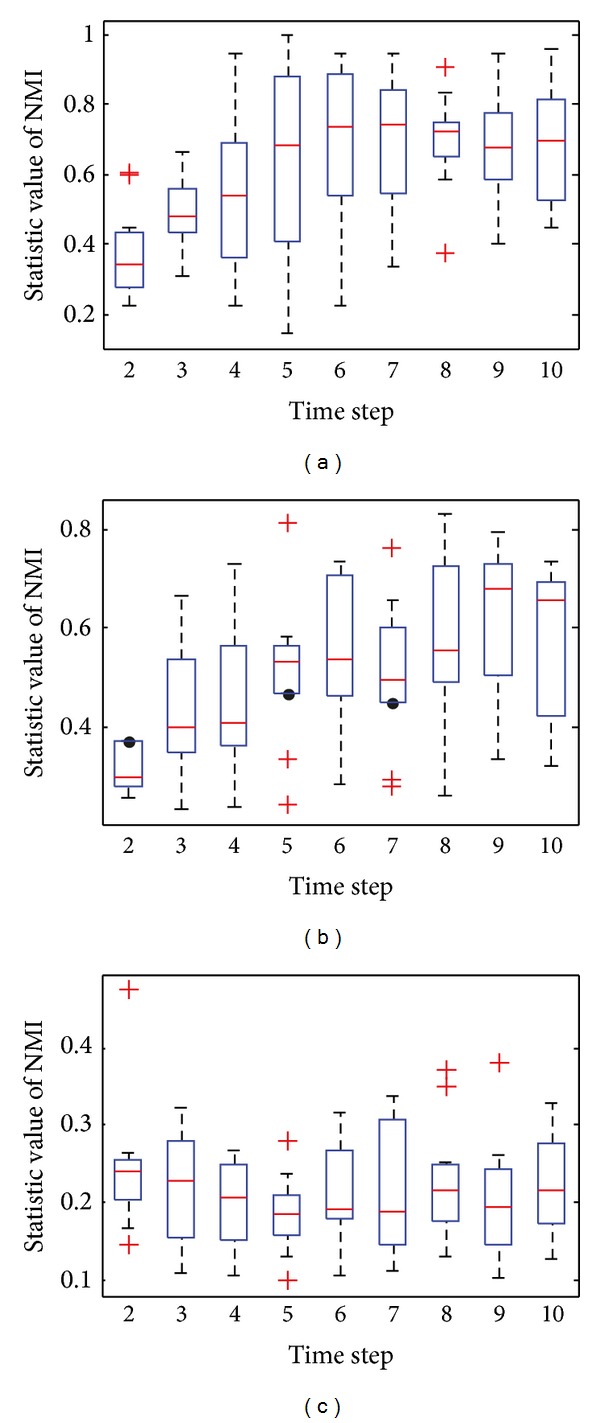
The box plot to illustrate the distribution of NMI at each time step when *z* = 8. (a) The box plot for DYN-DMLS; (b) the box plot for DYN-DMEA; (c) the box plot for DYN-MOGA.

Figure 10 shows the box plots to illustrate the distribution of the value of NMI when *z* = 8. Consensus clustering is not included for having no results. Three MOEAs are all of high variation ranging from bottom to top. It can be seen clearly the result of DYN-MOGA is stable but not good enough and that of DYN-DMEA could hardly reach more than 0.8. Therefore, DYN-DMLS is still the best in the three algorithms.

#### 4.1.2. Results on SYN-VAR

In this study, we generate the SYN-VAR datasets under four different levels by setting *z* = 5, 6,7, 8, where the community structures gradually change from clear to fuzzy. As introduced before, SYN-VAR contains the merging and splitting of communities. The dynamic process is more complex than SYN-FIX. Next the performance of the five algorithms on SYN-VAR would be checked.

**Figure 11:**
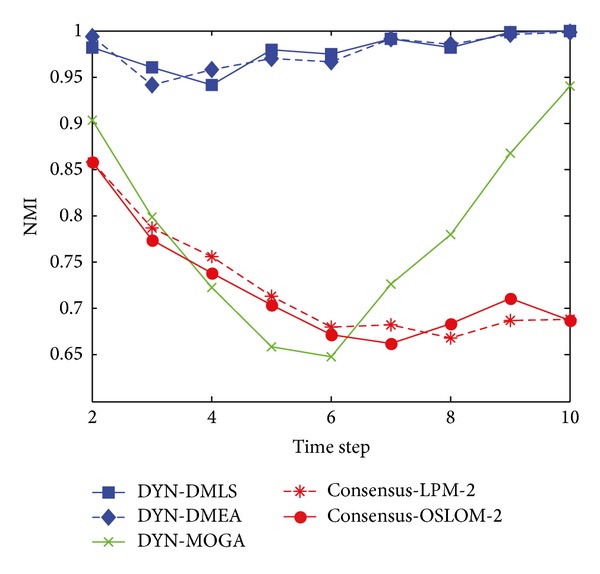
NMI results of the five algorithms on the SYN-VAR dataset with *z* = 5.

Figure 11 shows the statistical average value of NMI, when *z* = 5. DYN-DMLS and DYN-DMEA perform similarly well and a tiny promotion brought by local search could be observed. The curve of DYN-MOGA is much lower than the above two. The curve obtained by consensus clustering is low at most time at most time. Though the single run of LPM or OSLOM on the single time step network could result in the same with ground truth, the combination of the accurate structure could hardly satisfy neither of the involved time steps as the two structures differ much from each other. Consensus clustering may produce meaningful results, but it is not easy to prove by comparing with ground truth. However, MOEAs could do better under this evaluation system. Relatively speaking, DYN-DMLS could catch the merging and splitting of communities. More exactly, merging is traced more close than splitting as the second half of time steps, during which communities are merging, results better.

**Figure 12:**
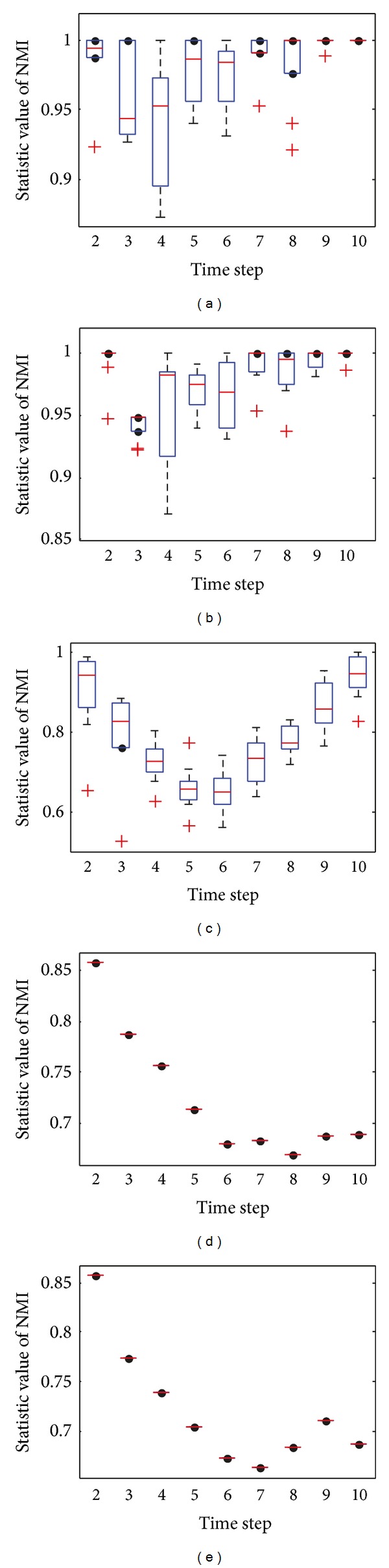
The box plots to illustrate the distribution of NMI at each time step on SYN-VAR when *z* = 5. (a) The box plot for DYN-DMLS; (b) the box plot for DYN-DMEA; (c) the box plot for DYN-MOGA; (d) the box plot for consensus with LPM; (e) the box plot for consensus with OSLOM.

Figure 12 shows the box plots to illustrate the distribution of the value of NMI on SYN-VAR when *z* = 5. The network with *z* = 5 is relatively simple but the stability does not seem well for such a simple network. As the dynamic becomes more complex than that in SYN-FIX, greater fluctuation is obtained by all the five algorithms. Consensus clustering always gets the same result because the static result of each time step keeps invariant in every repeat.

In SYN-VAR, every two consecutive time steps are less similar. It affects MOEAs more than consensus clustering. The merging and splitting of communities would lower the value of objective function NMI directly. When the objective is low, the number of probable solutions would increase. Generally speaking, it leads to a larger search space. Then, great fluctuation appears in the box plot. As to consensus algorithm, the deviation of two consecutive time steps would affect neither the separated static run at each time step nor the consensus process. But generally consensus clustering could not obtain a satisfying result.

**Figure 13:**
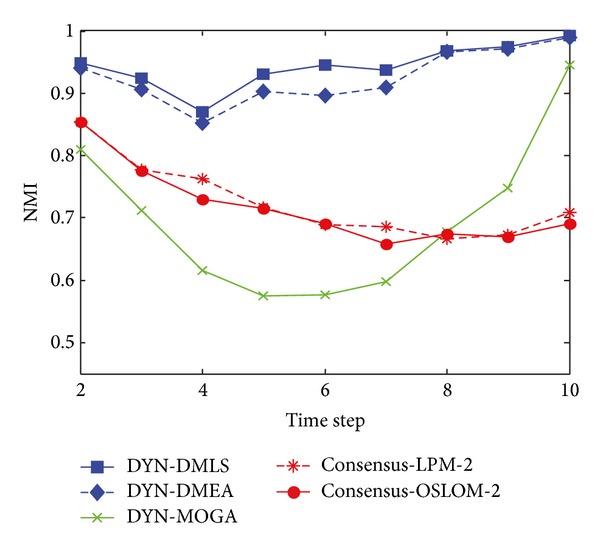
NMI results of the five algorithms on the SYN-VAR dataset with *z* = 6.

**Figure 14:**
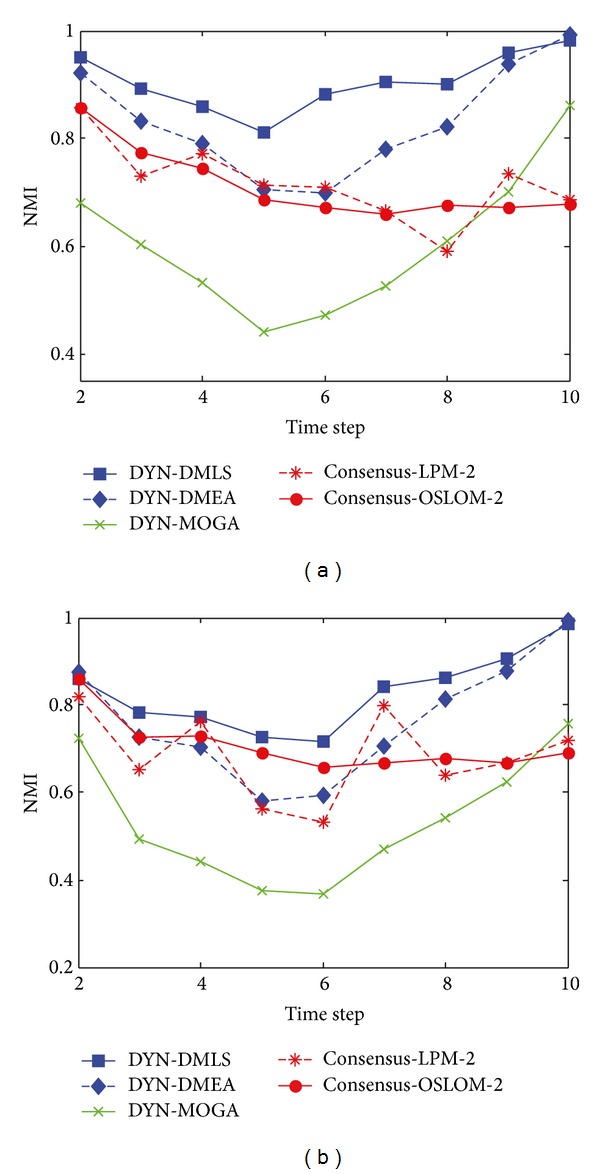
NMI results of the five algorithms on the SYN-VAR dataset with *z* = 7 and 8.

**Figure 15:**
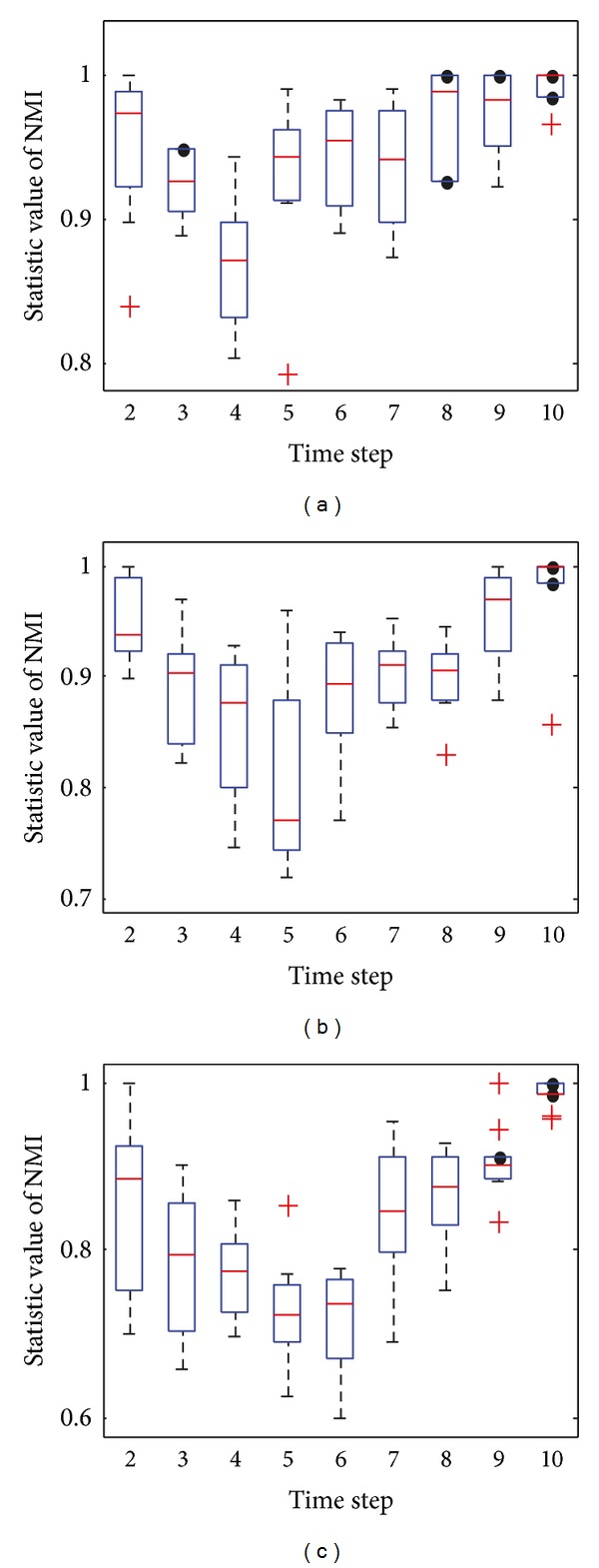
The box plots to illustrate the NMI distribution of DYN-DMLS at each time step on SYN-VAR when *z* = 6,7, 8.

Figure 13 shows the statistical average value of NMI when *z* = 6.  Figure 14 shows results with *z* = 7 and 8. These curves reflect the same tendency. DYN-DMLS here outperforms other algorithms obviously. Even when *z* = 8, a high NMI value close to 1 is obtained. The others fail to do so. Consensus clustering can work on SYN-VAR too. When *z* = 7 or 8 networks are complex, it still can provide a satisfying result, better than DYN-MOGA but worse than DYN-DMLS and DYN-DMEA.  Figure 15 shows the box plots of DYN-DMLS and consensus-OSLOM-2 to illustrate the distribution of the value of NMI on SYN-VAR when *z* = 6,7, 8. Other algorithms are ignored here. The two are enough to lead to the conclusion.

Overall considering the experimental results on synthetic networks, we can conclude the following.

- On most of the tested synthetic networks, DYN-DMLS performs best. Without local search process, DYN-DMEA always results in being a little lower than DYN-DMLS. This proves that the local search is effective. However, DYN-MOGA performs poor. These indicate the rationality of our method.
- Consensus clustering is good at finding the consensus part from a set of structures. Its result may be meaningful. But to our evaluation metric, its performance is not so good. Consensus clustering is of strong convergence. As we can see from the box plot, repeats bring about the same result. Because of the strong convergence and the stable results from static algorithms, the results may be rough and have no chance to be improved. The most important question is that which structure should be regarded as the corresponding ground truth for comparison. The evaluation metric of consensus clustering on dynamic network is tough.

### 4.2. Experiments on Real-Life Datasets

In this section, we present experimental studies on three real-life datasets: the football network dataset (http://www.jhowell.net/cf/scores/scoresindex.htm), the VAST dataset (http://www.cs.umd.edu/hcil/VASTchallenge08), and the DBLP Coauthorship Dataset (http://www.informatik.uni-trier.de/~ley/db/).

#### 4.2.1. Results on Football Network Dataset

The football network dataset is the National Collegiate Athletic Association (NCAA) Football Division 1-A Schedule, which has been used by Newman and Girvan [2]. The NCAA divides 116 schools into eleven conferences and games are more frequent between members of the same conference; in addition, there are four independent schools: Army, Brigham Young, Navy, and Notre Dame, which are grouped into one conference, but they have no more games with members of the same conference than that of the other conference. Nodes in the graph represent teams and edges represent the regular season games between the two teams. In our study, we select the years 2005–2009 to evaluate the performance of our algorithm, each year as a snapshot graph. Therefore, we investigate the football match network over the 5 time steps and there are 12 conferences and 120 teams at each time step. Because there is a priori knowledge about the ground truth of the community structure in the football network, we still employ NMI to evaluate the performance of our algorithm.

Note that the football dataset is dynamic. Though the ground truth is invariant, the network is always changing. The edges between nodes are changing. At each timestamp, it is not prior knowledge that the division is the same. It could be considered as a kind of strong time smoothing information. If the previous result is accurate enough, time smoothing would contribute more to getting a satisfying result for the current. This is not against the dynamic assumption but a probable situation.

Figure 17 shows the statistical average value of NMI with respect to the ground truth over time. It can be clearly seen that both DYN-DMLS and DYN-DMEA algorithms have much better performance than DYN-MOGA, while DYN-DMLS outperforms the DYN-DMEA. The average value of NMI obtained by DYN-DMLS is around 0.9 at each time step, which demonstrates that DYN-DMLS can find community structure accurately at each time step. However, consensus clustering is shining here. Both consensus-LPM-2 and consensus-OSLOM-2 could do better than DYN-DMLS. This is due to the fact that the football network has an invariant ground truth which gives it an environment similar to the static. So consensus clustering could produce a better result.

**Figure 16:**
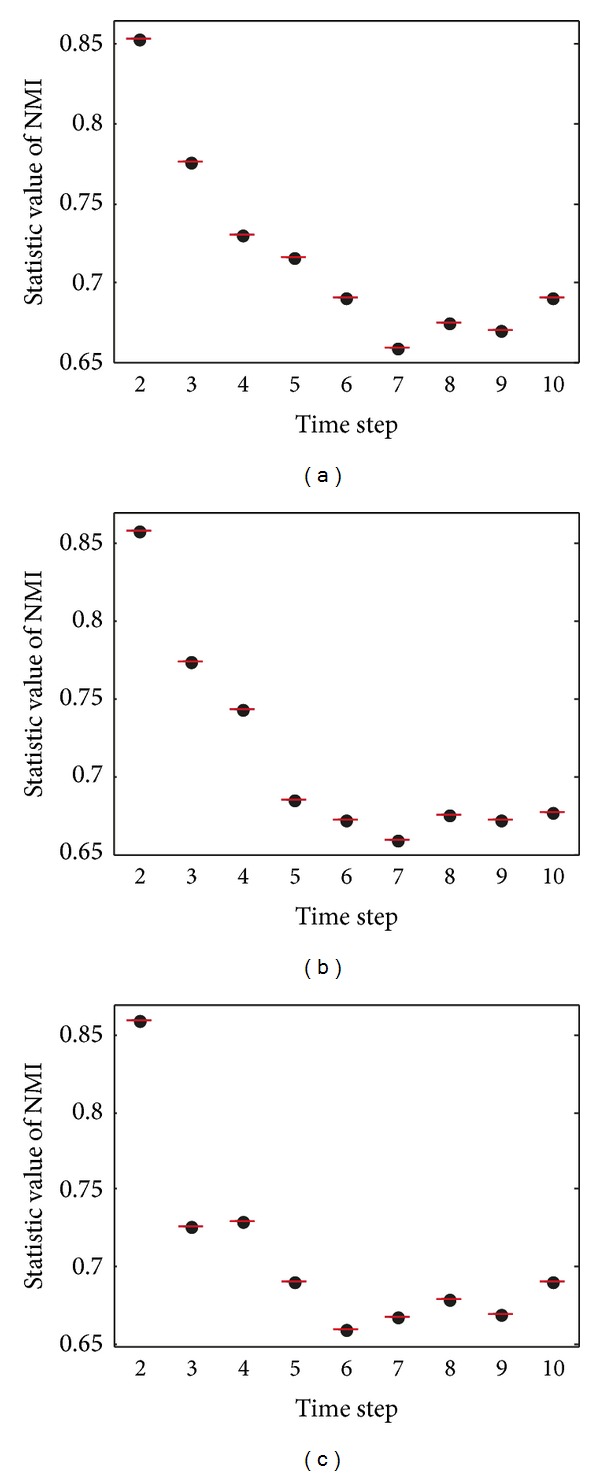
The box plots to illustrate the NMI distribution of consensus-OSLOM-2 at each time step on SYN-VAR when *z* = 6,7, 8.

**Figure 17:**
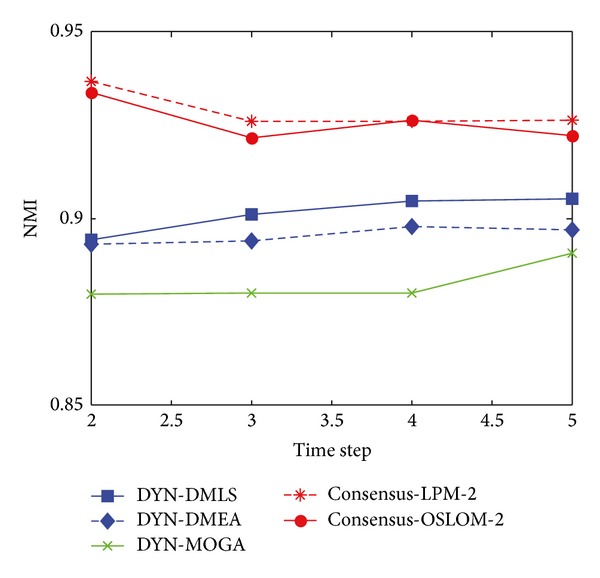
NMI results of the football dataset.

**Figure 18:**
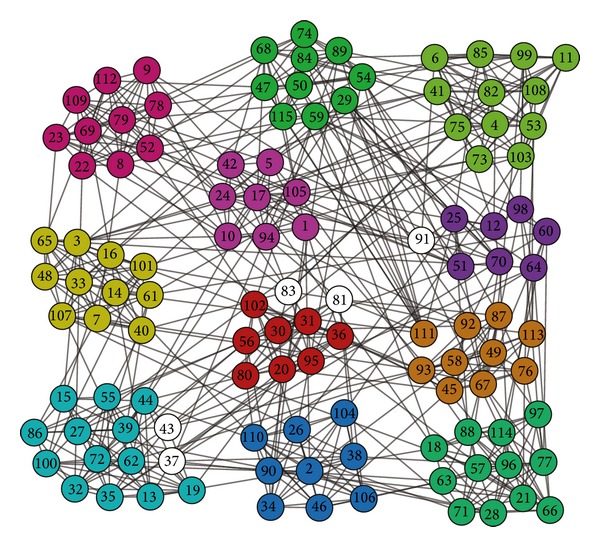
The communities found by DYN-DMLS on the football network for the year 2009.

In order to analyze visually, the communities found by our algorithm DYN-DMLS on the football network for the year 2009 are shown in Figure 18. The figure is obtained by using Pajek software [50]. The nodes with the same color denote that they belong to the same communities (i.e., conference). Particularly, we use 12 distinct RGB colors to label 12 true communities, which can be seen in Figure 16 in detail.

As what can be seen from Figure 18, DYN-DMLS can find 11 different communities. Almost all teams can be classified into true communities that they really belong to, which is an impossible mission for the other two algorithms. It can be clearly seen that only eight teams are mistakenly divided to the conferences Big 12, MAC, MWC, Pac 10, and WAC, respectively, which are shown in different colors in these 11 communities, where the teams of four independent schools, Army, Brigham Young, Navy, and Notre Dame, are included. The teams of the four independent schools should be in the same community according to the true communities partition, but they are divided into the other communities due to the fact that they have more frequent games with the teams in the other communities than between them. In a word, our algorithm can get better performance than the other two algorithms.

#### 4.2.2. Results on VAST Dataset

The VAST Dataset is a challenge task from IEEE VAST 2008, whose primary task is to characterize the Catalno/Vidro social network based on the cell phone call data provided and to characterize the temporal changes in the social structure over the 10-day period.

This dataset consists of information about 9834 calls between 400 cellphones over a 10-day period in June 2006 in the Isla Del Sueño. It includes records with the following fields: identifier for caller, identifier for receiver, time, duration, and call origination cell tower. In order to detect the communication patterns, we construct call graphs based on the call records. In order to evaluate our algorithm better, we convert the input social network and the corresponding dynamic graph into 5 snapshot graphs, where the graphs in every two days are aggregated into one snapshot graph and therefore we have 5 snapshot graphs over 10 days.

Note that the dataset records phone chains in 10 days. The phone call is a kind of temporary connection. It is in a short time window that the community structure would be confusing. Two members in the same community may make phone calls every day, while it is also common that there are just a few calls in the 10 days. One might not be able to catch the relationship at any time. To handle this kind of hidden information, time smoothing could work. As NMI is a kind of statistical information, little missing information would not affect the result too much. Therefore, time smoothing makes sense macroscopically.

Due to no a priori knowledge about ground truth of the cellphone network, the result has been figured out. Here, we only discover the community structure in the network to evaluate the performance of our algorithm, rather than performing the contest task which is the goal of the Mini Challenge 3.

**Figure 19:**
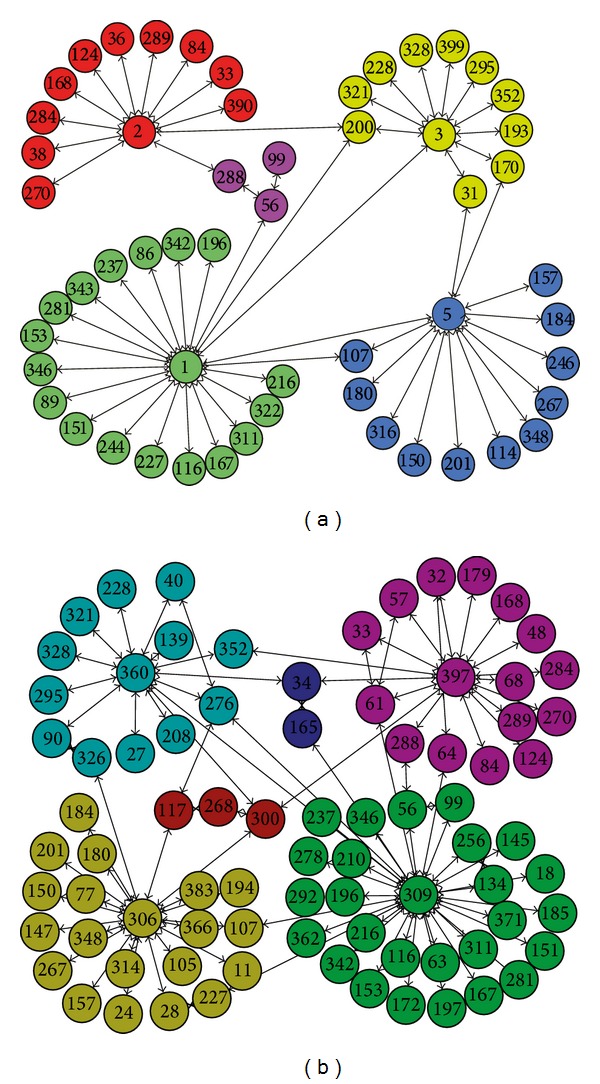
The main community structure of VAST found by DYN-DMLS at time step 3 and at time step 5. (a) The main community structure at time step 3; (b) the main community structure at time step 5.

As a challenge task from IEEE VAST 2008, this dataset has been analyzed by many researchers. It has been confirmed that the structure of the cellphone network changes drastically from the 7th day to the 8th day [51, 52]; that is, a significant variation happened at the high-level leaders during this period. Due to the 4th snapshot graph integrating the two graphs on the 7th and the 8th days, we display the main structure of the cellphone network at time step 3 and at time step 5 to analyze the changes before and after the event. It can be seen from Figure 19(a) that node 200 is the main leader, which contacts with nodes 1, 2, 3, and 5 during this time step, while these nodes are also group leaders in the Catalano hierarchy at time step 3. However, it can be clearly seen from Figure 19(b) that these nodes 300, 306, 309, 360, and 397 emerge as a new hierarchy at the time step 4. The community structure discovered by DYN-DMLS is consistent with the analysis that has been made.

#### 4.2.3. Results on DBLP Coauthorship Dataset

DBLP Coauthorship dataset is obtained from DBLP database, which has been described in [53]. In order to evaluate the performance of our algorithm, we choose a little part of DBLP data from the DBLP Coauthorship dataset to compose a connected graph. We select some authors from the DBLP database to form a small-scale DBLP Coauthorship dataset, which mainly focuses on the data mining areas. This dataset contains the coauthorship information among these papers over six years (2005–2010). By selecting authors who publish papers every year during the period of six years at these conferences including in DBLP database, 70 authors are chosen from the database to build connected snapshot graphs, where the nodes represent authors and edges represent coauthorship between two authors (nodes). The labels of these nodes corresponding to these authors are displayed in Table 1. For analyzing community evolutions, we aggregate data in every two years into one time step and therefore we have 3 time steps in total (corresponding to 3 snapshot graphs) for the dynamic network. In addition, we are not judging the quality or quantity of papers by an author. Instead, the importance of a node in a community is determined by its contribution to the community structure.

**Figure 20:**
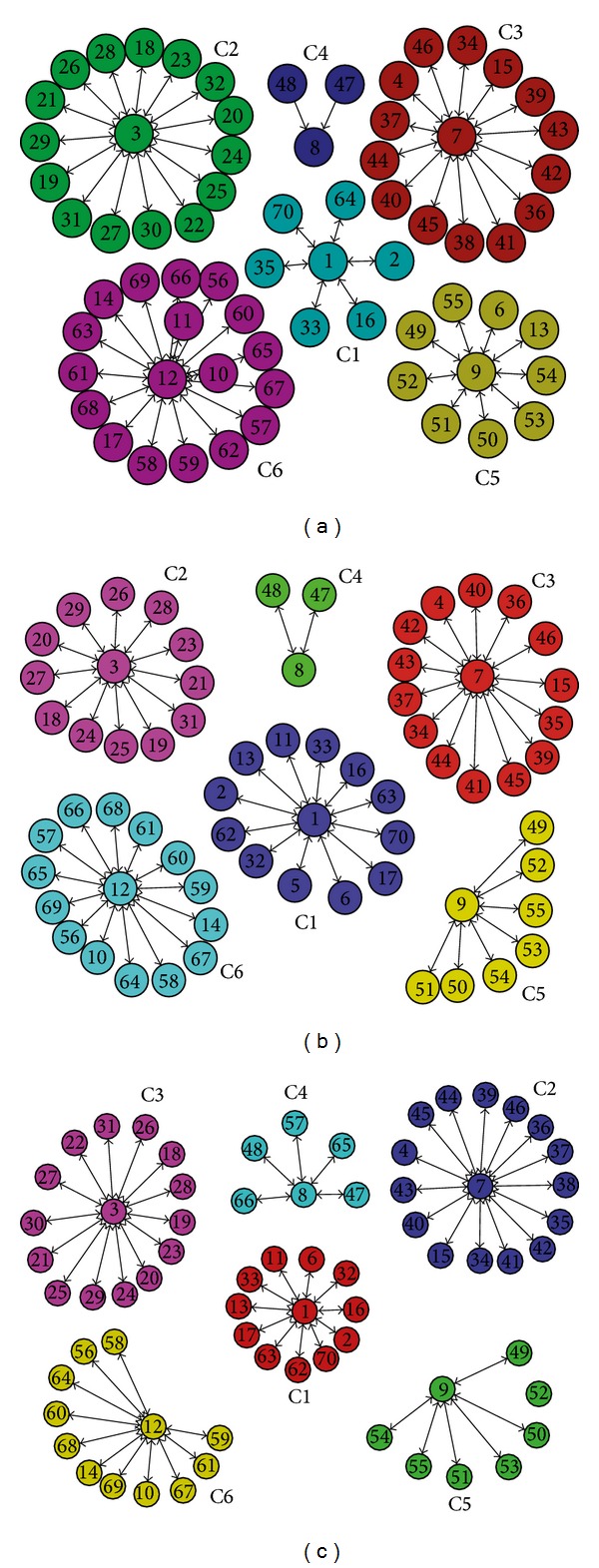
(a) The community partition on snapshot graph at first timestamp (2005-2006) without smooth evolution; (b) the community partition on snapshot graph at second timestamp (2007-2008) with smooth evolution; (c) the community partition on snapshot graph at third timestamp (2009-2010) with smooth evolution.

In this experiment, we apply our DYN-DMLS algorithm to analyze this dynamic network. Firstly, we detect the communities in snapshot graph at first time step (2005-2006) without smooth evolution by employing the memetic community detection algorithm (Meme-Net) [38] and the partition can be seen in Figure 20(a).

Then, DYN-DMLS algorithms are employed to detect the communities on the snapshot graphs over the other two time steps. Using the solution of our algorithm, we analyze how some individual authors' community membership changes over time.  Figure 20 shows the community partition on snapshot graph at each time step.

From Figure 20, we have the following observations. First, these partitions of the snapshot network at each time step can reflect the community structure well. Second, these partitions can reflect the temporal evolution well. For example, the nodes 6 and 13 are clustered into C5 at the first time step but are clustered into the C1 at the second and third time step; similarly, the nodes 11, 17, 62, and 63 also leave their original community and join the C1. In addition, the nodes 65, 66, and 57 are clustered into the C4 at the third time step, but they belong to the C6 at the first and the second time step. Therefore, the nodes divided into C1 and C4 over time reflect the temporal evolution well. The temporal evolution of the community structure is aroused by varying connection between these nodes; that is, the coauthorships among individual authors evolve over time. From the analyzing of these figures, we can find that the partition obtained by our algorithm corresponds with the ground truth of coauthorship among authors in these bibliographies in DBLP database.

### 4.3. The Effect of NMI as Time Smoothing

In our approach based on evolutionary clustering, NMI between the consecutive time steps is used to represent time smoothing. Time smoothing assumes that two consecutive time steps have structure connection with each other. Their community structures may not be the same but at least have a relatively higher similarity than that of two totally different ones. In evolutionary clustering, this corresponds to history cost (modularity corresponds to snapshot quality). NMI plays the role to measure the similarity. The higher the value of NMI is, the more similar the two consecutive time steps are. Therefore, its history cost is small. In the multiobjective optimization process, the solution which has high NMI but low modularity would be reserved in the population and it has a better chance to evolve into a proper solution than the one which has low NMI and modularity. Therefore, NMI can work as expected.

The experiment compares results of the two approaches on the benchmark network with *z* = 8. One is based on a single optimization of modularity which just calculates snapshot quality at every time step, and the other is our DYN-DMLS algorithm which simultaneously optimizes NMI and modularity (NMI represents time smoothing, while modularity represents snapshot quality).

**Figure 21:**
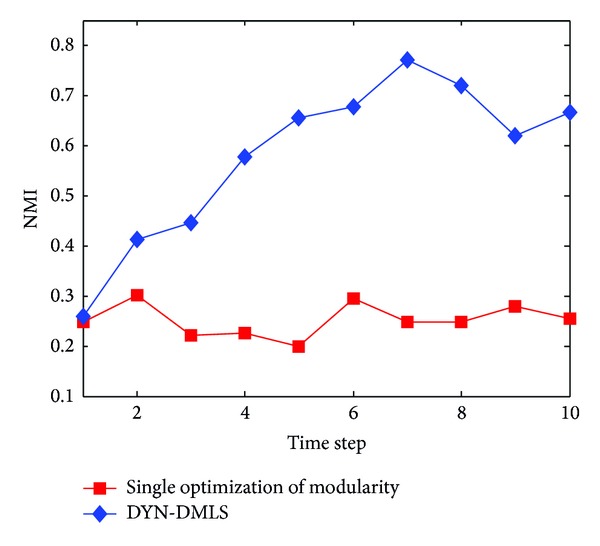
NMI results on the SYN-FIX with *z* = 8. Blue line represents the result of DYN-DMLS which simultaneously optimizes modularity and time smoothing. Red line represents the result of a single optimization of modularity at each time step.

When *z* = 8, networks are hard to cluster. In Figure 21, it is obvious that, in the complex situation (*z* = 8), single optimization of modularity could hardly improve the result. With extra consideration on time smoothing, higher NMI (NMI here is evaluation metric which is different from objective function as time smoothing) results are obtained. It also shows that the proposed method brings about a significant improvement by simultaneously optimizing snapshot quality (modularity) and history cost (NMI). Therefore, it proves the effectiveness of NMI as time smoothing.

### 4.4. Time Symmetry of DYN-DMLS

Though the result of the first time step has an influence on the subsequent process, the influence is rather limited and diminishing. When calculating objective function NMI, the result is directly determined by the two consecutive time steps, the current one and the previous one. The *t*th time step may directly affect the (*t* + 1)th but can hardly affect the (*t* + 2)th. To each single time step except the first one, it is always the previous one that determines its time smoothing. It is hard to quantify the diminishing influence. Besides, there is still a weighted parameter to balance between history cost and snapshot quality. Time smoothing as history cost could hardly contribute more to the result. Therefore, the first time step has not been given an excessive importance.

From another point of view, multiobjective optimization is a global statistical search process. Each step may generate different results in different runs. So time symmetric may be a property of the dynamic network but in multiobjective optimization it is hard to maintain.

**Figure 22:**
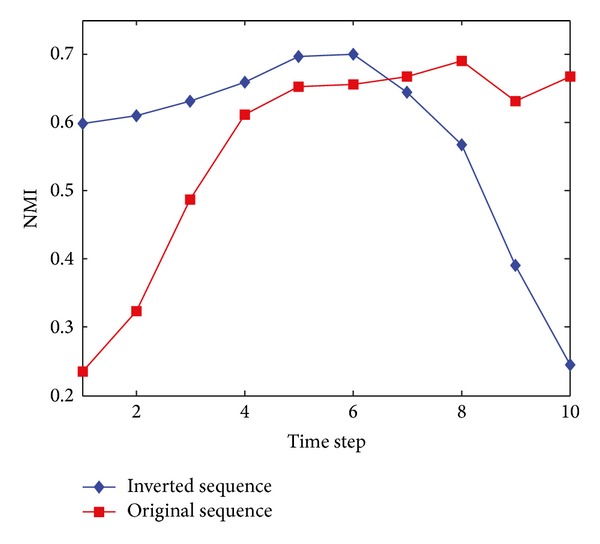
NMI results of the two sequences on the SYN-FIX with *z* = 8. Red line represents the result of a common and original sequence from 1 to 10, while blue line represents the result of an inverted sequence from 10 to 1. Method applied is DYN-DMLS.

To support the above description, we test the dynamic network in an inverted time sequence and compare its result with the common one. Strictly speaking, it is not symmetric. The result is shown in Figure 22. It is the improvement to the result that makes it look symmetric. The figure also tells that the first time step has not been given an excessive importance.

## 5. Concluding Remarks

The detection of communities and analysis of the community evolution in dynamic networks with temporal smoothness is a new challenging research problem with broad applications. In this paper, the two cost functions, community quality function and temporal cost function, are optimized simultaneously by the decomposition-based multiobjective evolutionary algorithm with a local search. The methods can provide the solution representing the best tradeoff between the accuracy of the communities structures obtained and the similarity between one time step and the previous one, without fixing a weight parameter in advance. In addition, a local search operator is incorporated into our method according to the problem-specific knowledge, which has a better ability to search the solution, especially when the community structure changes more dramatically over time. Experiments on SYN-VAR benchmark demonstrate that the proposed algorithm has a better accuracy in extracting community and capturing community evolution than the classic DYN-MOGA and consensus clustering algorithm. In our future work, we will expand our algorithm to be suitable for processing the large-scale networks in real life. Some better local search strategies should be studied to incorporate into our method to improve the performance further.

## Figures and Tables

**Algorithm 1:**
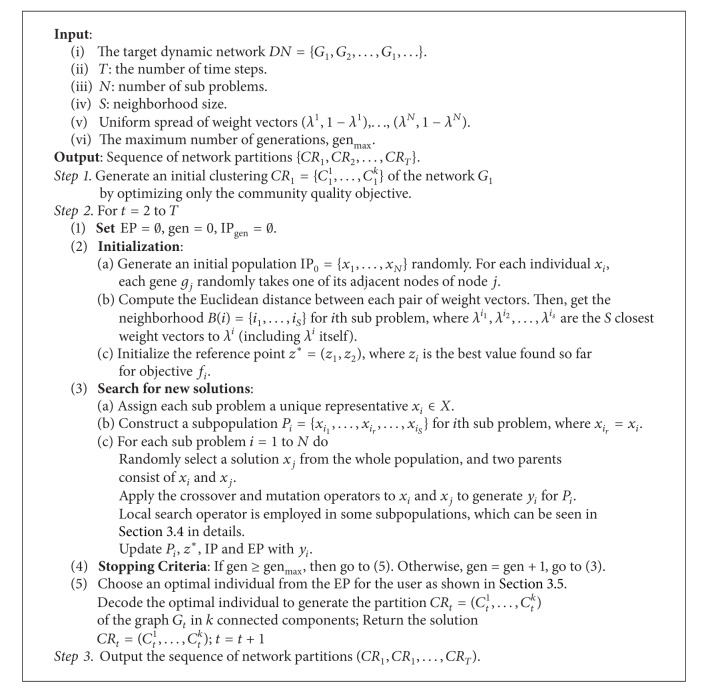
Framework of the MOEA/D-based community detection algorithm.

**Algorithm 2:**
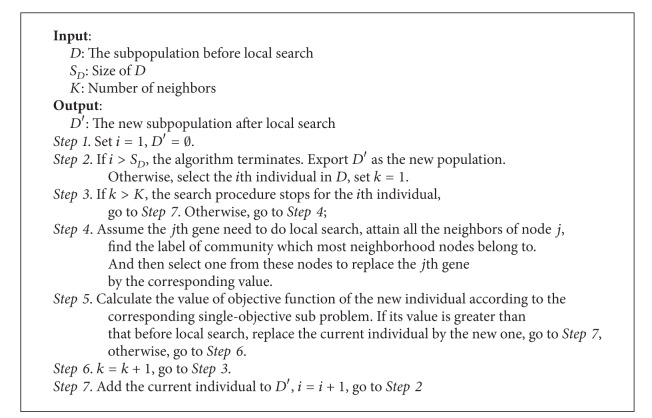
Local search procedure.

**Table 1:** The author corresponding to the nodes in snapshot graphs.

1 “P. S. Yu”	2 “C. C. Aggarwal”	3 “M. S. Chen”	4 “W. Fan”	5 “B. Gedik”	6 “J. Han”
7 “B. Liu”	8 “L. Liu”	9 “J. Pei”	10 “H.X. Wang”	11 “K. Wang”	12 “K. L. Wu”
13 “Y. Xu”	14 “X. F. Yan”	15 “Z. F Zhang”	16 “J.Y. Wang”	17 “C. C. Chen”	18 “H. L. Chen”
19 “M. C. Chen”	20 “W. T. Chen”	21 “Y. H. Chu”	22 “K.T. Chuang”	23 “J. M. Ho”	24 “J.H. Hsiao”
25 “C. M. Hsu”	26 “J. W. Huang”	27 “H. P. Hung”	28 “K. H. Liu”	29 “W. G. Teng”	30 “C. Y. Tseng”
31 “M. Y. Yeh”	32 “K. Zhang”	33 “L. Liu”	34 “D. Cai”	35 “C. Chen”	36 “H. Gonzalez”
37 “X. F. He”	38 “S. K. Kim”	39 “X. L. Li”	40 “H.Y. Liu”	41 “Q. Z. Mei”	42 “Z. Shao”
43 “D. Xin”	44 “X. X. Yin”	45 “C. X. Zhai”	46 “F.D. Zhu”	47 “N. J.”	48 “X. l. Li”
49 “J. Caverlee”	50 “K. K. Chen”	51 “A. Iyengar”	52 “C. Pu”	53 “A. Singh”	54 “M. Srivatsa”
55 “J. Yin”	56 “A. W. C. Fu”	57 “D. X. Jiang”	58 “X.M. Lin”	59 “Y. F. Tao”	60 “R. C. W. Wong”
61 “X. K. Xiao”	62 “X. m. Lin”	63 “X. F Meng”	64 “C. Zaniolo”	65 “B. C. M. Fung”	66 “E. P. Lim”
67 “H. W. Lauw”	68 “J.N. K. Liu”	69 “W. M. Ma”	70 “R. She”		
